# Reclassification of *Saccharomycodes sinensis*, Proposal of *Yueomyces sinensis* gen. nov., comb. nov. within Saccharomycetaceae (Saccharomycetales, Saccharomycotina)

**DOI:** 10.1371/journal.pone.0136987

**Published:** 2015-09-16

**Authors:** Long Wang, Marizeth Groenewald, Qi-Ming Wang, Teun Boekhout

**Affiliations:** 1 State Key Laboratory of Mycology, Institute of Microbiology, Chinese Academy of Sciences, Beijing, China; 2 CBS-KNAW Fungal Biodiversity Centre, Yeast Division, Utrecht, The Netherlands; Leibniz Institute DSMZ-German Collection of Microorganisms and Cell Cultures, GERMANY

## Abstract

The phylogenetic position of *Saccharomycodes sinensis* has been debated by yeast taxonomists. In this study, a multigene phylogenetic analysis based on four regions, namely the 18S ribosomal DNA (rDNA), the D1/D2 domains of the 26S rDNA, the second largest subunit of RNA polymerase II gene (*RPB2*) and translation elongation factor 1-α gene (*EF1-α*), were performed to address the phylogenetic placement of *S*. *sinensis*. Our result indicated that *S*. *sinensis* belongs to Saccharomycetaceae instead of Saccharomycodaceae, and forms a single species lineage divergent from the other genera within Saccharomycetaceae. *Yueomyces* gen. nov. (MycoBank No. MB 811648) is proposed in the Saccharomycetaceae with *Y*. *sinensis* comb. nov. (MycoBank No. MB 811649, type strain CGMCC 2.01395^T^ = IFO 10111^T^ = CBS 7075^T^) as the type species.

## Introduction

Saccharomycetaceae and Saccharomycodaceae are regarded as sister families within Saccharomycetales (Saccharomycetes, Saccharomycotina) [1, 2]. The latter family includes three genera, i. e. *Hanseniaspora*, *Saccharomycodes* and *Kloeckera*, which is the anamorphic counterpart of *Hanseniaspora* [3]. *Saccharomycodes* consists of two species, namely *S*. *ludwigii*, the type species of *Saccharomycodes*, and *S*. *sinensis*. The latter species was found to be phylogenetically divergent from the former one according to Yamada et al. [4] based on the sequence analysis of 18S ribosomal DNA (rDNA). Kurtzman and Robnett demonstrated that *S*. *sinensis* NRRL Y-12797^T^ had an identical sequence of the D1/D2 domains of the 26S rDNA with *Nadsonia fulvescens* var. *elongata* [5], so this species was not accepted in *Saccharomycodes* and treated as a synonym of *N*. *fulvescens* var. *elongata* [6]. Yamazaki et al. [7] showed that *S*. *sinensis* IFO10111^T^ had different sequences from *N*. *fulvescens* var. *elongata* in the D1/D2 domains of the 26S rDNA and ITS (ITS1+5.8S rDNA+ITS2) regions. The phenotypic characters of this strain were consistent with those of the original species description [8]. As a consequence of these conflicting results, the *S*. *sinensis* ex-type strain NRRL Y-12797 deposited in the ARS Culture Collection, Northern Regional Research Center, US Department of Agriculture, Peoria, USA, was re-examined and found to be different from the ex-type strain IFO 10111 deposited in the Institute for Fermentation, Osaka, Japan [9]. The sequence analysis of the D1/D2 domains of the 26S rDNA by Yamazaki et al. [7] indicated that *S*. *sinensis* did not belong to any known genera within Saccharomycodaceae. Boundy-Mills et al. [10] indicated that this species was not a member of *Saccharomycodes*, but suggested that additional gene sequence analyses were needed to be sure about its phylogenetic placement. In present study, the ex-type strain of *S*. *sinensis* HS 506 (= CGMCC 2.01395) and the holotype AS 2.1395 originally described by Yue [8] and deposited in the China General Microbiological Culture Collection Center (CGMCC), Institute of Microbiology, Chinese Academy of Sciences, Beijing, China, and the ex-type strain CBS 7075 deposited in CBS Fungal Biodiversity Centre (CBS-KNAW), Utrecht, The Netherlands, were examined to clarify the phylogenetic placement of this species based on a multigene analysis, including the 18S rDNA, the D1/D2 domains of 26S rDNA, the second largest subunit of RNA polymerase II gene (*RPB2*) and translation elongation factor 1-α gene (*EF1-α*).

## Materials and Methods

### Strains

The holotype of *Saccharomycodes sinensis* AS 2.1395 and the ex-type strain HS 506 (= CGMCC 2.01395) deposited in the China General Microbiological Culture Collection Center (CGMCC), Institute of Microbiology, Chinese Academy of Sciences, Beijing, China, and the ex-type strain CBS 7075 deposited in CBS Fungal Biodiversity Centre (CBS-KNAW), Utrecht, The Netherlands, were used in this study.

### Molecular phylogenetic analysis

Genomic DNA was extracted using the method as described by Wang and Bai [11]. The rDNA sequences, including the ITS (ITS1+5.8S+ITS2), the D1/D2 domains of 26S rDNA and the 18S rDNA, and the *RPB2* and the *EF1*-*α* gene sequences were determined according to Wang et al. [12]. Sequences were aligned with the MUSCLE program in MEGA 5 [13] and the sequences were concatenated for analysis. All sites including gaps in the concatenated dataset were used for phylogenetic analysis. The models of nucleotide substitution were investigated in MEGA 5 and the general time-reversible model of DNA substitution that assumes a percentage of invariable sites and Γ-distributed substitution rates at the remaining sites (GTR + I + G) was suggested as the best-fit nucleotide substitution model. Thus, this model was selected for Maximum likelihood (ML) analysis conducted in MEGA 5 using 1000 bootstrap replicates analysis. Maximum parsimony (MP) analysis was conducted in MEGA 5 from 1000 replicates using 10 random additions and TBR for each replicate. Bayesian inference (BI) was conducted in MrBayes 3.2 [14] with GTR + I + G model and 2000000 generations, and all other parameters were settled according to previous study [15]. Two independent runs and four chains started with random trees. Trees were sampled every 1000 generations leading to an overall sampling of 2000 trees. The analysis was stopped when the standard deviation of split frequencies between the trees generated in the independent runs was below 0.01. The first twenty-five percent of these trees were discarded, the remaining were used to compute a 50% majority rule consensus tree to obtain estimates for posterior probabilities. The GenBank accession numbers obtained in this study are KP866224–KP866232 and KP866234–KP866246. The other sequences used here were retrieved from GenBank (S1 Table).

## Results and Discussion

The holotype of *S*. *sinensis* AS 2.1395 and the ex-type strain of *S*. *sinensis* HS 506 (= CGMCC 2.01395) have identical ITS and D1/D2 sequences with the ex-type strain IFO10111 (AB056132 and AB127389). The ITS and D1/D2 sequences of CBS 7075, ex-type strain of *S*. *sinensis*, are the same as that of IFO10111 (http://www.cbs.knaw.nl/Collections/Biolomics.aspx?Table=CBS+strain+database). The D1/D2 sequences of AS 2.1395 and CBS 7075 differ from those of strain NRRL Y-12797 (U94946). The latter NRRL strain has an identical sequence with *Nadsonia fulvescens* var. *elongata* (U94942).

**Fig 1:**
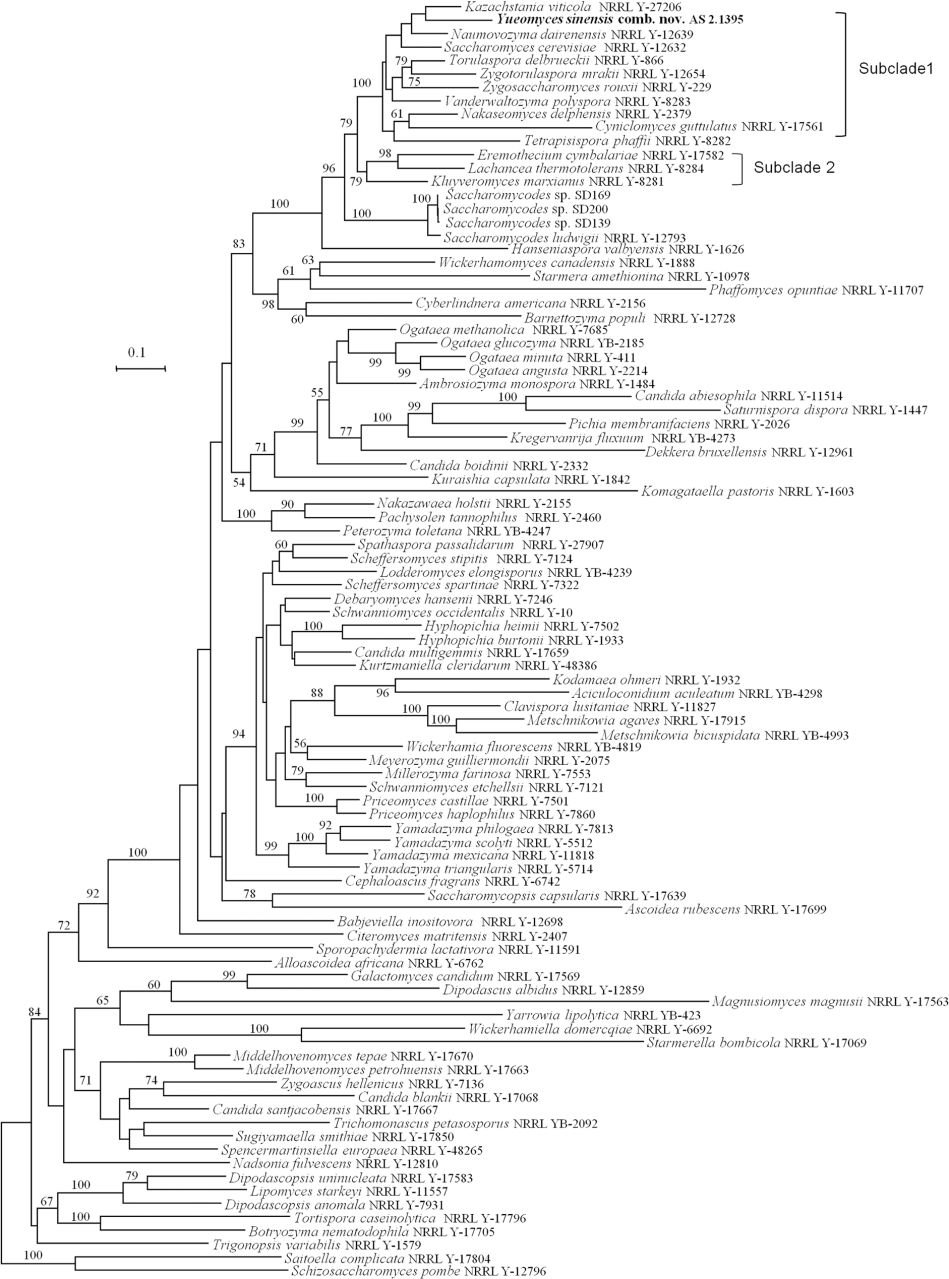
The multigene ML tree. Phylogenetic tree constructed from Maximum likelihood (ML) analysis of the combined sequences of the18S rDNA, D1/D2 domains of the 26S rDNA, the *RPB2* and *EF1-α*, depicting *Yueomyces sinensis* and other taxa in the Saccharomycetales (Saccharomycotina). Bootstrap percentages greater than 50% from 1000 bootstrap replicates are shown. Bar = 0.1 indicates 10% sequence divergence.

The multigene phylogenetic study for assessing the relationships among ascomycetous yeast genera (Saccharomycotina, Ascomycota) published by Kurtzman and Robnett [2], including the type species of currently recognized genera within Saccharomycotina, indicated the presence of 12 evolutionary clades. Saccharomycetaceae and Saccharomycodaceae were sister families and located in the largest clade of Saccharomycotina [2]. However, the phylogenetic placement of *S*. *sinensis* was not addressed in their study. Here we attempted to investigate the sequence diversity of six gene fragments, the 18S rDNA, the D1/D2 domains of the 26S rDNA, the ITS (ITS1+5.8S rDNA+ITS2), the two RNA polymerase II genes (*RPB1* and *RPB2*) and the translation elongation factor 1-α gene (*EF1-α*), to clarify the phylogenetic position of *S*. *sinensis*. We failed to get PCR amplicons of the *RPB1* gene. A phylogenetic tree (Fig 1) using the combined *RPB2*, *EF1-α*,18S rDNA and D1/D2 domains of the 26S rDNA genes was constructed, including the taxa employed in the study of Kurtzman and Robnett [2]. Our result showed that *S*. *sinensis* was quite divergent from the species of *Saccharomycodes* (Saccharomycodaceae), but was closely related to the species within Saccharomycetaceae (Fig 1). *S*. *sinensis* was found to be related to the genus *Kazachstania* but this relationship lacked bootstrap support in the ML tree (Fig 1), to *Tetrapisispora* in the MP tree (S1 Fig), and had an unresolved relationship with some genera of Saccharomycetaceae (i.e. *Kazachstania*, *Naumovozyma*, *Saccharomyces* and *Tetrapisispora*) in the BI tree (S2 Fig). Although *S*. *sinensis* formed a single species lineage distinct from the other genera in the Saccharomycetaceae using the above three methods of analysis, it occurred in Subclade 1 that was delimited by *Kazachstania viticola* and *Tetrapisispora phaffii* (Fig 1), by *Saccharomyces cerevisiae* and *Zygotorulaspora mrakii* (S1 Fig), and by *S*. *sinensis* and *Cyniclomyces guttulatus* (S2 Fig). These relationships were found to be supported in each tree (ML support 100, MP support 92, BI support 0.94). The genera *Eremothecium*, *Kluyveromyces* and *Lachancea* formed Subclade 2 in the Saccharomycetaceae (Fig 1, S1 and S2 Figs) which is in agreement with Kurtzman and Robnett [2]. The cells of *S*. *sinensis* are lemon-shaped (apiculate) with bipolar budding similar to those of species within Saccharomycodaceae [8], whereas the other species of the genera in the Saccharomycetaceae form ellipsoid, ovoid to cylindrical cells with multilateral budding [3]. We concluded that *S*. *sinensis* represents a new genus in the Saccharomycetaceae, and *Yueomyces* gen. nov. is proposed to accommodate this species.

The topology of Clade 1 as defined in Kurtzman and Robnett [2] had some differences in comparison to our analysis (Fig 1). *Vanderwaltozyma polyspora and Tetrapisispora phaffii* clustered together, but lacked bootstrap support in Kurtzman and Robnett [2], whereas the two species separated into two groups in our study (Fig 1). Incongruences between trees obtained from different datasets have been observed [1]. Five genes, namely *RPB1*, *RPB2*, *EF1-α*, 18S rDNA and 26S rDNA, were used in the study of Kurtzman and Robnett [2], whereas *RPB1* gene was not included in our analysis due to the lack of this gene sequence for *S*. *sinensis*. The phylogenetic discordance between our study and that of Kurtzman and Robnett [2] may be due to the different datasets used. Another difference between the study of Kurtzman and Robnett [2] and ours, is that the former did not contain gapped positions in contrast to the current study.

Saccharomycodaceae was established by Kudryavtsev [16] and is characterized by the presence of apiculate cells with bipolar budding. This family includes four ascomycetous genera, namely *Hanseniaspora*, *Nadsonia*, *Saccharomycodes* and *Wickerhamia* [16–19]. *Hanseniaspora* and *Saccharomycodes* are closely related to the genera in the Saccharomycetaceae based on single gene and multigene sequence analyses [1, 2, 20]. The genera *Nadsonia* and *Wickerhamia* are well separated from Saccharomycodaceae as well as from one another based on the sequence analysis of the D1/D2 domains of the 26S rDNA [21]. However, a recent phylogenetic study indicated that *Wickerhamia* might be related to *Lodderomyces* [2] and was placed in the family Debaryomycetaceae [3, 21]. *Nadsonia* appeared to be closely related to *Ascoidea* in the tree drawn from the D1/D2 domains of the 26S rDNA [5, 9], however, this was not supported by multigene analysis [2]. *Nadsonia* was treated as *incertae sedis* in the Saccharomycetales [2, 3]. Thus, Saccharomycodaceae probably includes only the genera *Hanseniaspora* and *Saccharomycodes* that has only one species, *S*. *ludwigii*. The above studies showed that the presence of apiculate cells and bipolar budding are not the unique phylogenetic characters of the Saccharomycodaceae as suggested by Kurtzman [21], and Kurtzman and Robnett [2].

### Nomenclature

The electronic version of this article can be obtained in Portable Document Format (PDF) using the ISSN or ISBN. This article represents a published work according to the International Code of Nomenclature for algae, fungi, and plants [22]. The new names contained in the electronic publication of a PLOS ONE article are effectively published under this nomenclature code from the electronic edition alone; there is therefore no longer any need to provide printed copies.

In addition, the new names contained in this work have been submitted to MycoBank, from where they will be made available to the Global Names Index. The unique MycoBank number can be resolved and the associated information viewed through any standard web browser by appending the MycoBank number contained in this publication to the prefix http://www.mycobank.org/MB/. The online version of this work is archived and available from the following digital repositories: PubMed Central and LOCKSS.

#### Description of *Yueomyces* Q.M. Wang, L. Wang, M. Groenewald & T. Boekhout gen. nov

[urn:lsid:indexfungorum.org:names: 811648], MycoBank MB 811648

Etymology: The genus is named in honor of Jing-Zhu Yue for her contributions to the taxonomy of yeasts in China.

Member of Saccharomycetaceae (Saccharomycetales, Saccharomycotina). This genus is mainly circumscribed by the multigene phylogenetic analysis in which it forms an isolated position in the Saccharomycetaceae (Fig 1, S1 and S2 Figs). Colonies are cream, butyrous, smooth, and with entire margins. The yeast cells are lemon-shaped (apiculate) and reproduce by bipolar budding. Asci contain one to four spherical ascospores with a smooth surface. Starch-like compounds are not produced. Glucose and galactose are fermented. Nitrate is not assimilated. The major ubiquinone is Q-6 [4].

Type species: *Yueomyces sinensis* (J.Z. Yue) Q.M. Wang, L. Wang, M. Groenewald & T. Boekhout comb. nov. MycoBank MB 811649.

Basionym: *Saccharomycodes sinensis* J.Z. Yue, Acta Microbiol. Sin. 17: 91. 1977.

#### The morphological and physiological data of *Yueomyces sinensis* [8]

In yeast extract-malt extract broth (YM broth), after 3 days at 28°C, the cells are ellipsoidal and lemon-shaped (apiculate), 2.5–6.0 × 3.3–10.0 μm, and occur singly or in pairs. Budding is bipolar. After 1 month at 28°C, a sediment and ring are present. After 1 month at 28°C, the streak culture is cream-colored, smooth, and the margin is entire. Pseudohyphae are not observed on corn-meal agar. Sporulation is observed on Gorodkowa agar and Kleyn’s acetate agar after 5–7 days at 28°C. Ascus formation occurs without prior conjugation, and asci usually contain one to two or rarely three or four spherical ascospores with a smooth surface. Glucose and galactose are fermented, but sucrose, melibiose, raffinose, maltose, and lactose are not fermented. Glucose, galactose, and glucolactone are assimilated. Sucrose, L-sorbose, maltose, lactose, trehalose, D-ribose, raffinose, inulin, melibiose, melezitose, cellobiose, soluble starch, D-xylose, L-arabinose, D-arabinose, L-rhamnose, D-glucosamine, methanol, ethanol, erythritol, ribitol, galactitol, glycerol, D-mannitol, α-methyl-D-glucoside, DL-lactic acid, succinic acid, citric acid, salicin, inositol, and hexadecane are not assimilated. Ammonium sulfate, ethylamine hydrochloride, cadaverine hydrochloride, L-lysine, potassium nitrate, and sodium nitrite are not assimilated, but casamino acid is assimilated. Starch-like compounds are not produced. Growth in vitamin-free medium is negative. The type strain, HS 506^T^, was isolated from soil, Mount Chienfeng, Hainan Island, China, in September 1974. This strain has been deposited in the China General Microbiological Culture Collection Center (CGMCC), Institute of Microbiology, Chinese Academy of Sciences, Beijing, China, as CGMCC 2.01395^T^, in the Institute for Fermentation, Osaka, Japan, as IFO 10111^T^, and in CBS Fungal Biodiversity Centre (CBS-KNAW), Utrecht, The Netherlands, as CBS 7075^T^. The holotype AS 2.1395^T^, dried culture of HS 506^T^, has been deposited in CGMCC, Institute of Microbiology, Chinese Academy of Sciences, Beijing, China.

Note: the strain NRRL Y-12797 deposited in the ARS Culture Collection, Northern Regional Research Center, US Department of Agriculture, Peoria, USA, does not represent the ex-type of *S*. *sinensis* because it seems to be replaced by an isolate of *N*. *fulvescens* var. *elongata*.

## Supporting Information

S1 Data New sequence data. The new sequences of *Yueomyces sinensis* and *Saccharomycodes* sp.
(TXT)

S1 Fig The MP tree. Phylogenetic tree constructed from Maximum parsimony (MP) analysis of the combined sequences of the18S rDNA, D1/D2 domains of the 26S rDNA, the *RPB2* and *EF1-α*, depicting *Yueomyces sinensis* and other taxa relationships in the Saccharomycetales (Saccharomycotina). Bootstrap percentages greater than 50% from 1000 bootstrap replicates are shown.
(TIF)

S2 Fig The BI tree. Phylogenetic tree constructed from Bayesian inference (BI) analysis of the combined sequences of the18S rDNA, D1/D2 domains of the 26S rDNA, the *RPB2* and *EF1-α*, depicting *Yueomyces sinensis* and other taxa relationships in the Saccharomycetales (Saccharomycotina). Bayesian posterior probabilities above 0.9 are shown. Bar = 0.1 indicates 10% sequence divergence.
(TIF)

S1 Table GenBank numbers The sequences GenBank numbers retrieved from GenBank database. (XLS)
