# Supplementary figures and images for: Reclassification of Saccharomycodes sinensis, Proposal of Yueomyces sinensis gen. nov., comb. nov. within Saccharomycetaceae (Saccharomycetales, Saccharomycotina)
Source: PLoS One. 2015 Sep 16;10(9):e0136987. doi: 10.1371/journal.pone.0136987 (PMC4573984; doi:10.1371/journal.pone.0136987)

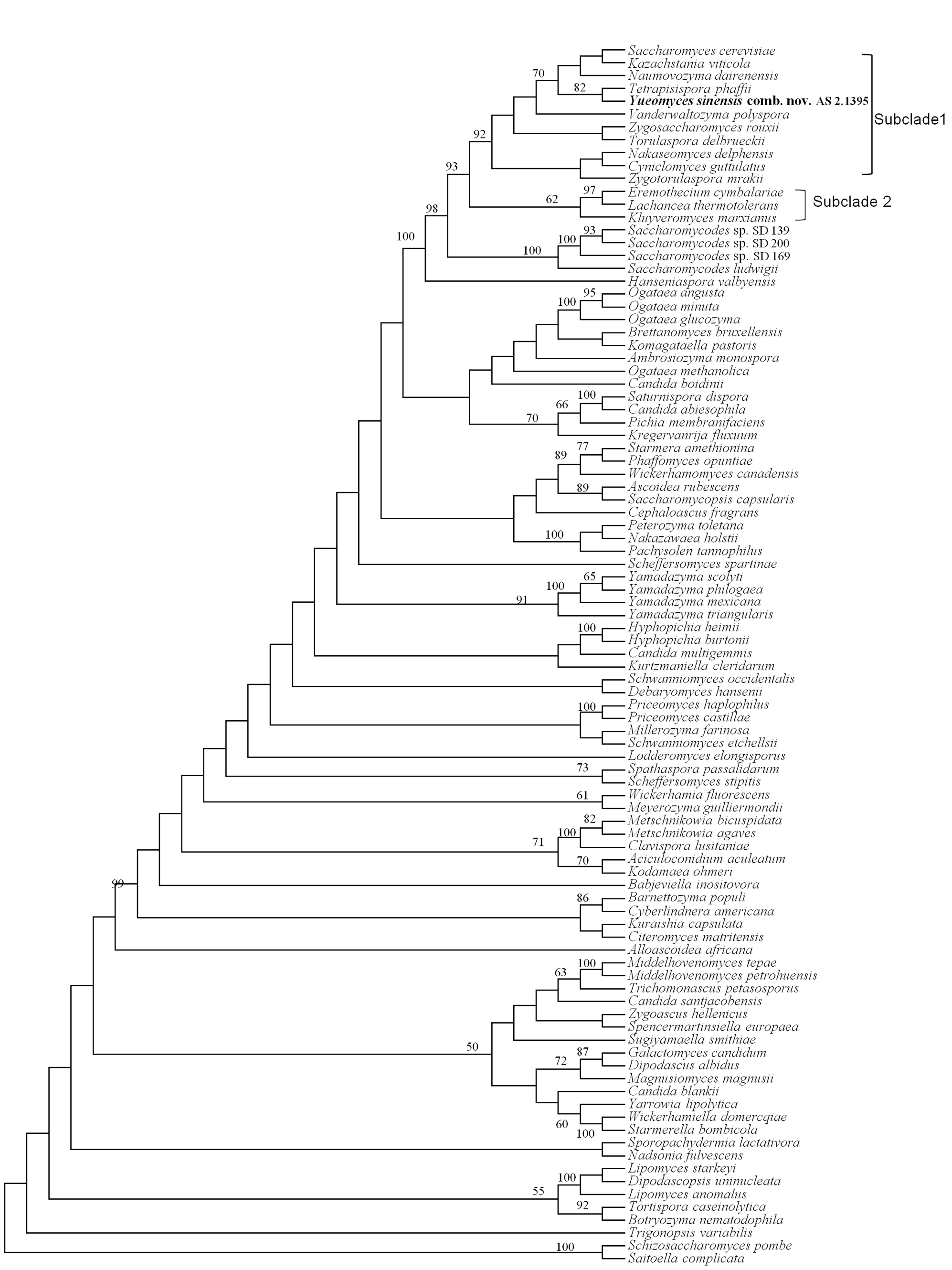

Supplement: S1 Fig — Phylogenetic tree constructed from Maximum parsimony (MP) analysis of the combined sequences of the18S rDNA, D1/D2 domains of the 26S rDNA, the RPB2 and EF1-α, depicting Yueomyces sinensis and other taxa relationships in the Saccharomycetales (Saccharomycotina). Bootstrap percentages greater than 50% from 1000 bootstrap replicates are shown. (TIF) [file pone.0136987.s002.tif]

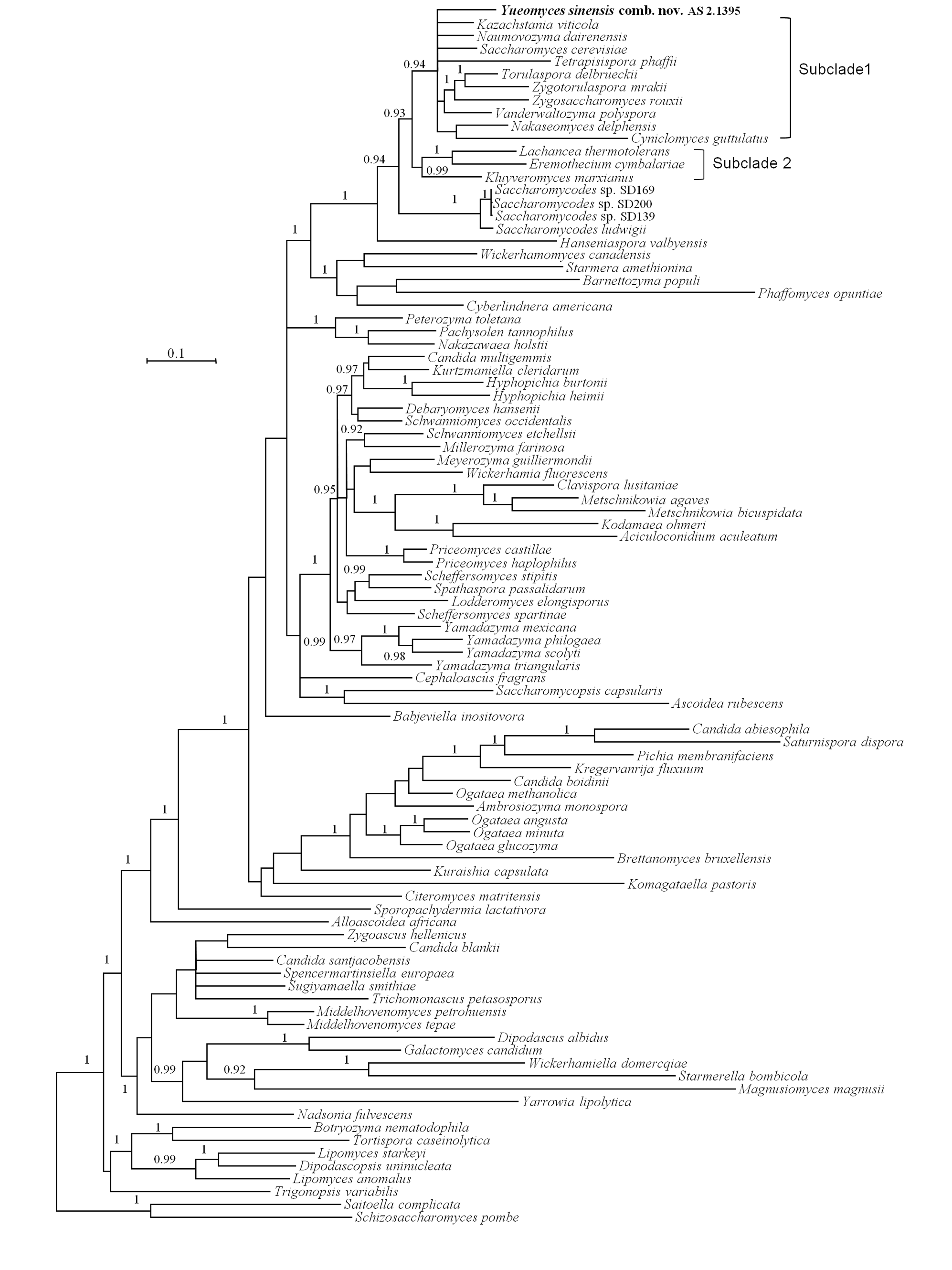

Supplement: S2 Fig — Phylogenetic tree constructed from Bayesian inference (BI) analysis of the combined sequences of the18S rDNA, D1/D2 domains of the 26S rDNA, the RPB2 and EF1-α, depicting Yueomyces sinensis and other taxa relationships in the Saccharomycetales (Saccharomycotina). Bayesian posterior probabilities above 0.9 are shown. Bar = 0.1 indicates 10% sequence divergence. (TIF) [file pone.0136987.s003.tif]
